# Alteration of swing leg work and power during human accelerated sprinting

**DOI:** 10.1242/bio.024281

**Published:** 2017-04-10

**Authors:** Ryu Nagahara, Takeo Matsubayashi, Akifumi Matsuo, Koji Zushi

**Affiliations:** 1 National Institute of Fitness and Sports in Kanoya, 1 Shiromizu-cho, Kanoya, Kagoshima 891-2393, Japan; 2 Japan Institute of Sports Sciences, 3-15-1 Nishigaoka, Kita, Tokyo 115-0056, Japan; 3 Faculty of Health and Sport Sciences, University of Tsukuba, 1-1-1 Tennoudai, Tsukuba, Ibaraki 305-8574, Japan

**Keywords:** Kinetics, Acceleration, Running, Locomotion, Lower-extremity

## Abstract

This study investigated changes in lower-extremity joint work and power during the swing phase in a maximal accelerated sprinting. Twelve male sprinters performed 60 m maximal sprints while motion data was recorded. Lower-extremity joint work and power during the swing phase of each stride for both legs were calculated. Positive hip and negative knee work (≈4.3 and ≈−2.9 J kg^−1^) and mean power (≈13.4 and ≈−8.7 W kg^−1^) during the entire swing phase stabilized or decreased after the 26.2±1.1 (9.69±0.25 m s^−1^) or 34.3±1.5 m mark (9.97±0.26 m s^−1^) during the acceleration phase. In contrast, the hip negative work and mean power during the early swing phase (≈7-fold and ≈3.7-fold increase in total), as well as the knee negative work and power during the terminal swing phase (≈1.85-fold and ≈2-fold increase in total), increased until maximal speed. Moreover, only the magnitudes of increases in negative work and mean power at hip and knee joints during the swing phase were positively associated with the increment of running speed from the middle of acceleration phase. These findings indicate that the roles of energy generation and absorption at the hip and knee joints shift around the middle of the acceleration phase as energy generation and absorption at the hip during the late swing phase and at the knee during early swing phase are generally maintained or decreased, and negative work and power at hip during the early swing phase and at knee during the terminal swing phase may be responsible for increasing running speed when approaching maximal speed.

## Abstract (precis)

**Summary:** The roles of respective swing leg joints intricately change with increases in running speed during accelerated sprinting.

## INTRODUCTION

Accelerated running, which means running with acceleration or deceleration (negative acceleration), is more common than steady speed running in daily life. However, a limited number of studies have investigated the biomechanics of human accelerated running (e.g. Biewener and Daley, 2007; Hunter et al., 2004; Johnson and Buckley, 2001; Morin et al., 2015, Nagahara et al., 2014a,b; Rabita et al., 2015; Segers et al., 2007, 2014; Van Caekenberghe et al., 2013). During human accelerated running, particularly the acceleration phase of sprinting, a deep hanging posture of trunk becomes upright, and the angular velocities of the lower-extremity joints increase across multiple steps as running speed increases (Nagahara et al., 2014a). Therefore, an investigation of actual accelerated sprinting over a long distance (dozens of steps) is necessary to accurately understand the mechanisms of human accelerated sprinting and its changes.

Joint work and power have been studied to help understand energy generation and absorption at joints during locomotion in many species (Ae et al., 1987; Belli et al., 2002; Farris and Sawicki, 2012; McGowan et al., 2005; Rubenson and Marsh, 2009; Rubenson et al., 2011; Schache et al., 2011, 2015). Several studies have investigated changes in human lower-extremity joint work and power as steady running speed increases (Ae et al., 1987; Belli et al., 2002; Schache et al., 2011, 2015). For example, Schache et al. (2015) recently revealed that positive and negative knee joint work and power, as well as positive and negative ankle joint work, during the support phase increase until the middle (approximately 5 m s^−1^) of the range of steady running speeds from jogging to maximal speed (8.95 m s^−1^), and decrease or stabilize thereafter. Moreover, they also demonstrated that positive and negative hip joint work and power during the support and swing phases, as well as negative knee joint work and power during the swing phase, increase throughout the range of increase in running speeds (Schache et al., 2015). Based on these findings, Schache et al. (2015) concluded that the faster steady running speeds are not simply achieved by proportional increases in lower-extremity joint work and power. Although previous studies have provided fundamental knowledge of changes in joint work and power as steady running speed increases, alterations of joint work and power during accelerated sprinting have never been reported. It is likely that during accelerated sprinting changes in the amount of energy generation and absorption at the lower-extremity joints show different features to those seen at various steady speeds, because the profiles of changes in spatiotemporal and kinematic variables during acceleration phase of sprinting are different from those investigated at various steady speeds running (Belli et al., 2002; Dorn et al., 2012; Nagahara et al., 2014a,b; Novacheck, 1998). Therefore, further research is requisite to clarify how the human lower-extremity joints regulate work and power with increasing locomotion speed under acceleration conditions.

While joint work and power during the support phase of running are interesting and important characteristics for understanding running mechanics, attention has also been paid to joint work and power during the swing phase (Ae et al., 1987; Chapman and Caldwell, 1983; Dorn et al., 2012; Knuesel et al., 2005; Schache et al., 2011, 2015; Vardaxis and Hoshizaki, 1989). For example, Ae et al. (1987) showed that increases in steady running speeds correlate with positive hip joint work and negative knee joint work during the swing phase. The entire swing phase can be divided into several sub-phases based on the profile of changes in joint power at hip and knee (Schache et al., 2011). The lower-extremity decelerates before the ends of both forward and backward swing during sprinting and the swing velocity increases along with increment of running speed during the acceleration phase (from 0 to over 10 m s^−1^ of running speed), suggesting the large energy absorption before the end of forward or backward swing and the great extent of changes in amount of energy absorption at hip and knee during the acceleration phase of sprinting. Indeed, Chapman and Caldwell (1983) verified that negative knee joint power before foot strike limits achieving maximal speed. Accordingly, examining the amount of joint work and power during the entire and sub-phases of the swing phase could bring a detailed understanding of energy generation and absorption in the specific movements of lower-extremity in the acceleration phase of sprinting and has the potential to provide insight into the nature of the human locomotor system when accelerating. Recently, Nagahara et al. (2014a) verified that the magnitude (mean angular velocity during the support phase and range of flexion/extension) of hip joint movement gradually and slightly decreases from the 14th step (≈670° s^−1^ and ≈98° at ∼22 m mark) to maximal speed at around the 25th step (≈640° s^−1^ and ≈94° at ∼45 m mark) during the acceleration phase of sprinting. These findings suggest that some of the hip joint work and power variables during the swing phase decrease in the later acceleration phase in sprinting.

List of symbols

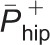
mean power generated by the hip during the swing phase
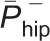
mean power absorbed by the hip during the swing phase
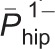
mean of the first power absorption by the hip during the swing phase
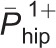
mean of the first power generation by the hip during the swing phase
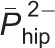
mean of the second power absorption by the hip during the swing phase
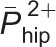
mean of the second power generation by the hip during the swing phase
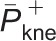
mean power generated by the knee during the swing phase
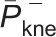
mean power absorbed by the knee during the swing phase
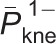
mean of the first power absorption by the knee during the swing phase
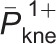
mean of the first power generation by the knee during the swing phase
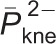
mean of the second power absorption by the knee during the swing phase
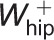
positive work done by the hip during the swing phase
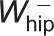
negative work done by the hip during the swing phase
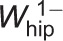
work done of the first power absorption by the hip during the swing phase
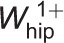
work done of the first power generation by the hip during the swing phase
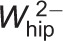
work done of the second power absorption by the hip during the swing phase
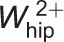
work done of the second power generation by the hip during the swing phase
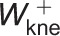
positive work done by the knee during the swing phase
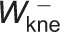
negative work done by the knee during the swing phase
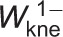
work done of the first power absorption by the knee during the swing phase
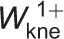
work done of the first power generation by the knee during the swing phase
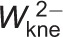
work done of the second power absorption by the knee during the swing phase
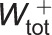
total positive work done by the lower-extremity during the swing phase
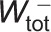
total negative work done by the lower-extremity during the swing phase

The present study aimed to demonstrate the alterations in lower-extremity joint work and power during the swing phase in the acceleration phase of maximal sprinting and to clarify whether the magnitudes of the joint work and power decrease when approaching maximal speed. We hypothesized that the changes in hip and knee joint work and power variables during the entire and sub-phases of the swing phase would show different profile during acceleration phase of sprinting (i.e. some variables do not increase until the maximal speed).

## RESULTS

**Fig. 1.**
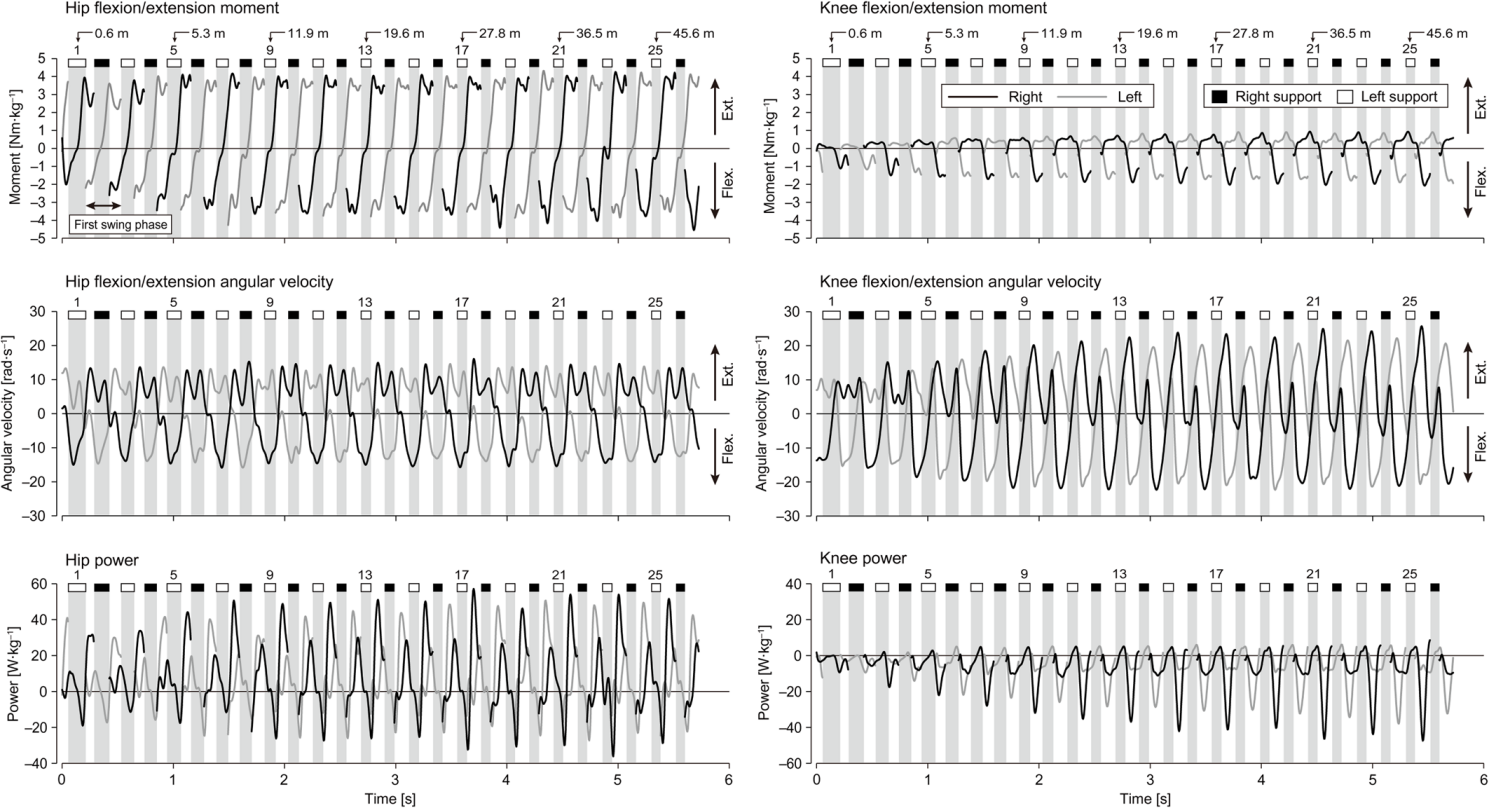
**Typical changes in hip and knee joint moment, angular velocity and power during the acceleration phase.** Grey backgrounds show support phases. Data are only shown in the sagittal plane.

**Fig. 2.**
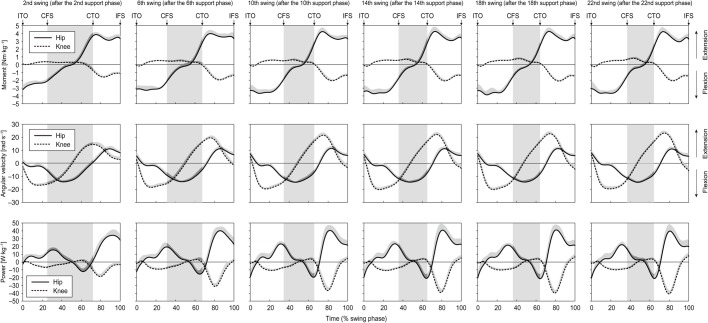
**Averaged changes in hip and knee joint moments, angular velocities and powers during swing phases after the 2nd, 6th, 10th, 14th, 18th and 22nd steps in the acceleration phase.** The top, middle and bottom rows show hip and knee moments in the sagittal plane, hip and knee angular velocities in the sagittal plane, and hip and knee joint power, respectively. Grey background shows contralateral support phase. ITO, ipsilateral toe-off; CFS, contralateral foot strike; CTO, contralateral toe-off; IFS, ipsilateral foot strike.

The fastest 60 m sprint time was 7.24±0.16 s. Figs 1 and 2 show that although the positive and negative peak values of the joint moments, angular velocities and powers during the swing phase generally increased over the entire acceleration phase except hip flexion/extension angular velocity, the phase profiles of these variables were approximately the same for all swing phases except the initial three or four steps. The running speed and distance from the starting line at the end of each section (4th, 8th, 12th, 16th, 20th, and 24th step) were 6.54±0.18 m s^−1^ at 4.7±0.3 m, 8.28±0.19 m s^−1^ at 11.0±0.5 m, 9.19±0.23 m s^−1^ at 18.3±0.8 m, 9.69±0.25 m s^−1^ at 26.2±1.1 m, 9.97±0.26 m s^−1^ at 34.3±1.5 m, and 10.03±0.28 m s^−1^ at 42.7±1.9 m.

### Changes in joint work and power during the entire swing phase in accelerated sprinting

**Fig. 3.**
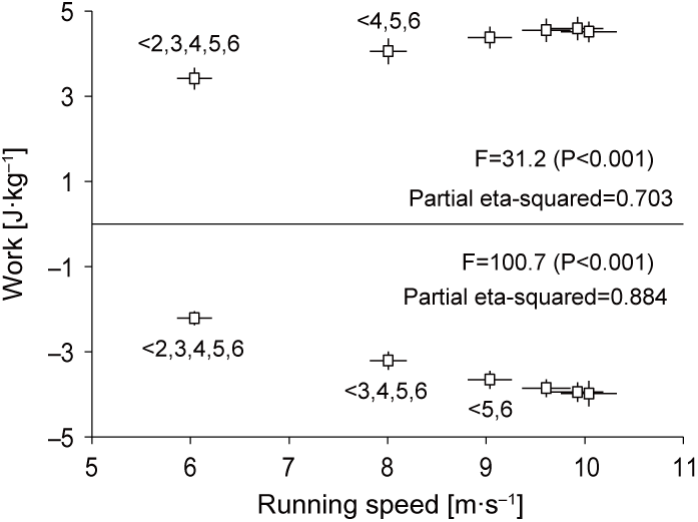
**Changes in total work generated and absorbed by the lower-extremity joints during the swing phase of accelerated sprinting.** Total work generation (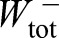) or absorption (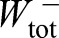) is the sum of positive or negative work done at the hip, knee and ankle joints during the entire swing phase. Values for all 24 swing phases were pooled into six sections (section 1 to 6) of swing phases (four swing phases per section), and these are presented as means±s.d. of 12 participants. The inequality signs with numbers indicate the results of the Bonferroni *post hoc* test.

Fig. 3 shows changes in total work generated and absorbed by the lower-extremity joints during the entire swing phase in acceleration phase of sprinting. The positive total work done by the leg during the swing phase 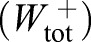 was initially 3.42±0.26 J kg^−1^, increasing by 34% by section 5 (4.59±0.27 J kg^−1^), and then decreased, while negative total work done by the leg during the swing phase 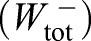 was initially −2.21±0.16 J kg^−1^ and increased ≈2-fold during the entire acceleration phase (−3.98±0.30 J kg^−1^ at section 6).

**Fig. 4.**
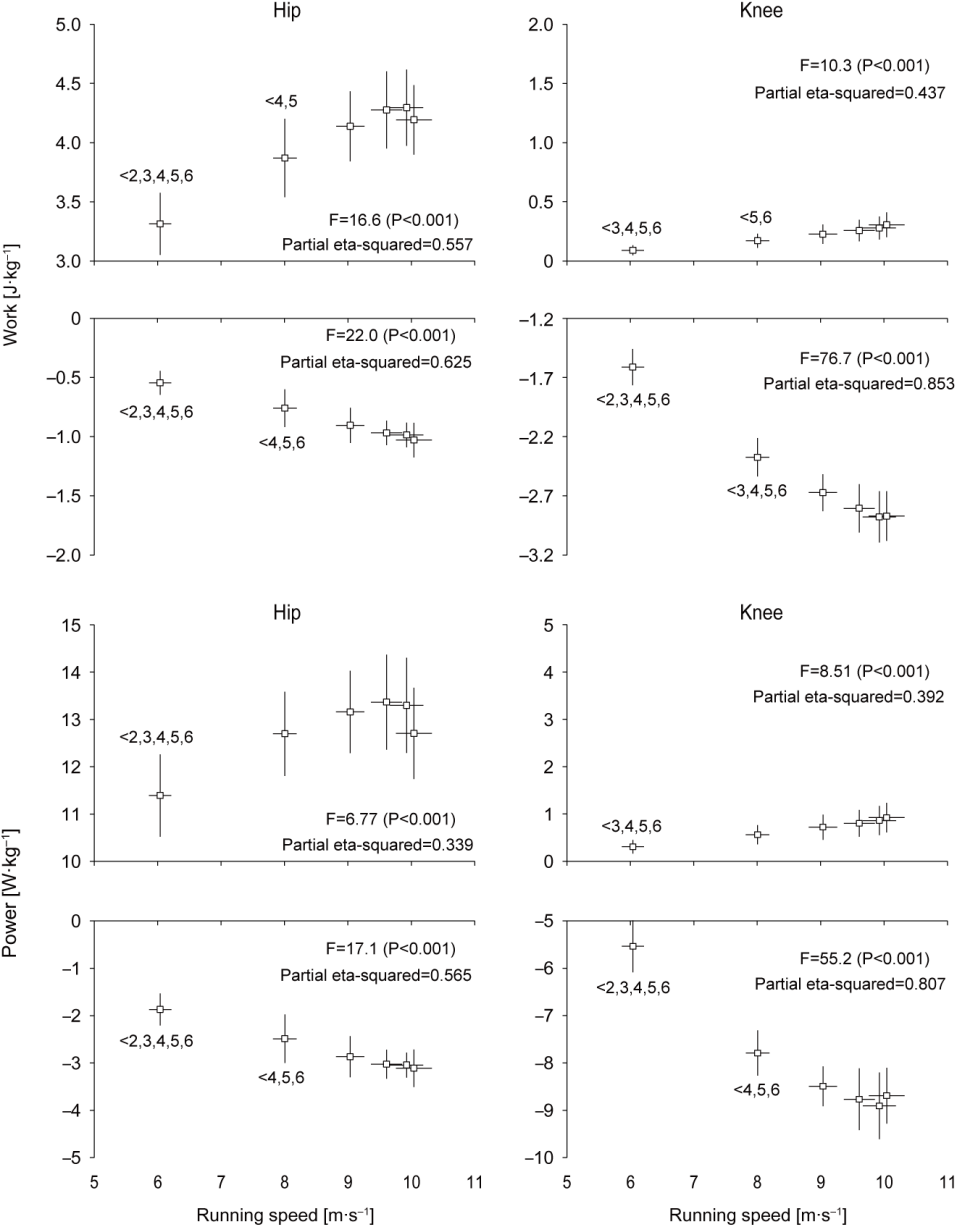
**Changes in work and mean power generated and absorbed by the hip and knee joints during the swing phase of accelerated sprinting.** The left and right panels show 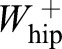 and 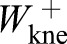, 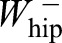 and 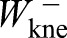, 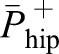 and 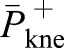, and 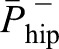 and 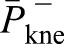, in top to bottom rows, respectively. Values for all 24 swing phases were pooled into six sections (section 1 to 6) of swing phases (four swing phases per section), and these are presented as means±s.d. of 12 participants. See Materials and Methods further detail. The inequality signs with numbers indicate the results of the Bonferroni *post hoc* test. Note the different *y*-axis scales for the panels.

Fig. 4 shows changes in work and mean power generated and absorbed by the hip and knee joints during the entire swing phase in acceleration phase of sprinting. The positive work done at the hip during swing phase 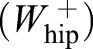 was 3.32±0.26 J kg^−1^ initially, increased by 30% by section 5 (4.30±0.32 J kg^−1^), and then slightly decreased. The negative work done at the hip during swing phase 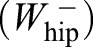 was initially −0.55±0.10 J kg^−1^ and increased gradually ≈2-fold with increased running speed until maximal speed was reached (−1.03±0.15 J kg^−1^). The positive work done at the knee during swing phase 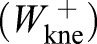 was initially 0.09±0.04 J kg^−1^ and slightly and gradually increased ≈3-fold with increasing the magnitude as running speed increased until maximal speed (0.31±0.11 J kg^−1^). The negative work done at the knee during swing phase 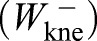 was initially −1.61±0.15 J kg^−1^, increased by 79% with decreasing magnitude by section 5 (−2.88±0.22 J kg^−1^), and slightly decreased afterward. Changes in mean power generation and absorption at the hip (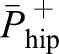, 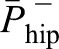) and knee (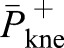 and 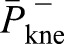) during the entire swing phase showed similar profiles to the corresponding joint work with a much clearer trend in the increases or decreases. The initial values were 11.4±0.9 W kg^−1^ for 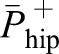, −1.9±0.3 W kg^−1^ for 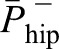, 0.3±0.1 W kg^−1^ for 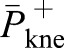 and −5.5±0.6 W kg^−1^ for 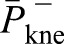, while the maximal values were 13.4±1.0 W kg^−1^ at section 4 for 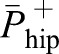, −3.1±0.4 W kg^−1^ at section 6 for 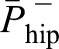, 0.9±0.3 W kg^−1^ at section 6 for 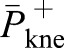 and −8.7±0.6 W kg^−1^ at section 5 for 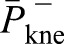.

### Changes in joint work and power during sub-phases of the swing phase in accelerated sprinting

**Fig. 5.**
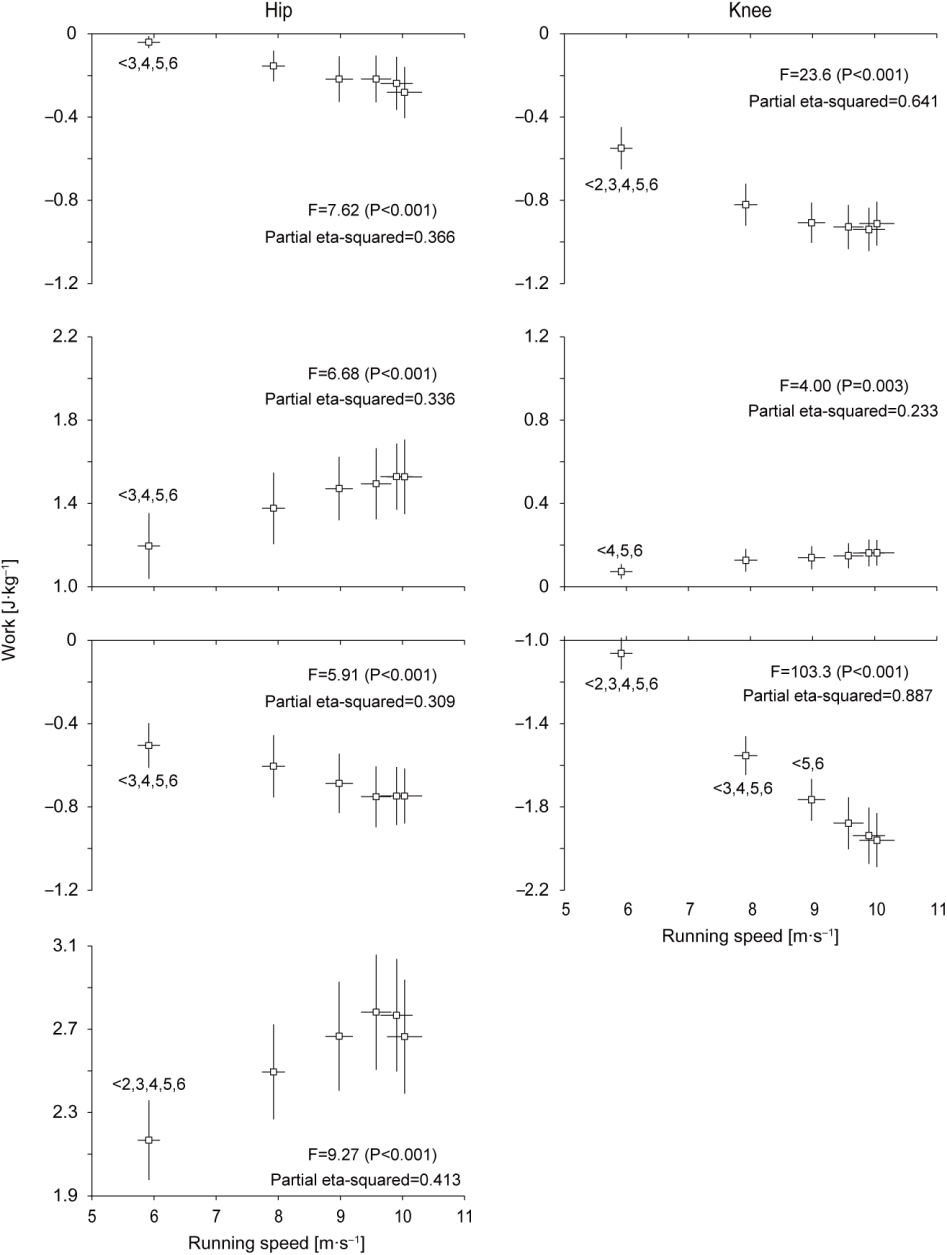
**Changes in work generated and absorbed by the hip and knee joints during the respective sub-phases of the entire swing phase of accelerated sprinting.** The left and right panels show 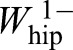 and 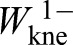, 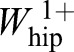 and 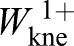, and 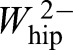 and 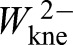, in top to the second lowest rows, respectively. The bottom left panel shows 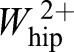. Values for all 24 swing phases were pooled into six sections (section 1 to 6) of swing phases (four swing phases per section), and these are presented as means±s.d. of 12 participants. See Materials and Methods for further detail. The inequality signs with numbers indicate the results of the Bonferroni *post hoc* test. Note the different *y*-axis scales for the panels.

Fig. 5 shows changes in work generated and absorbed by the hip and knee joints during the respective sub-phases of the entire swing phase in acceleration phase of sprinting. The first negative (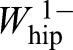) and positive hip work (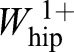) during the swing phase were initially −0.04±0.03 and 1.20±0.16 J kg^−1^, respectively, and increased 7-fold and 28%, respectively, during the entire acceleration phase (−0.28±0.12 and 1.53±0.18 J kg^−1^ at section 6). In contrast, the second negative (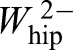) and positive hip work (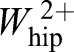) during the swing phase were initially −0.51±0.11 and 2.17±0.19 J kg^−1^ and increased by 47% and 28% by section 4 (−0.75±0.15 and 2.78±0.28 J kg^−1^), before 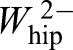 plateaued and 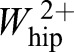 decreased. The first negative knee work (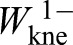) during the swing phase was initially −0.55±0.10 J kg^−1^, increased with decreasing magnitude until section 5 (−0.94±0.10 J kg^−1^), and then decreased. The first positive (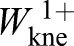) and the second negative knee work (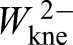) during the swing phase were initially 0.07±0.04 and −1.06±0.08 J kg^−1^, respectively, and linearly increased 2.3-fold and 85%, respectively, during the entire acceleration phase (0.16±0.06 and −1.96±0.13 J kg^−1^ at section 6).

**Fig. 6.**
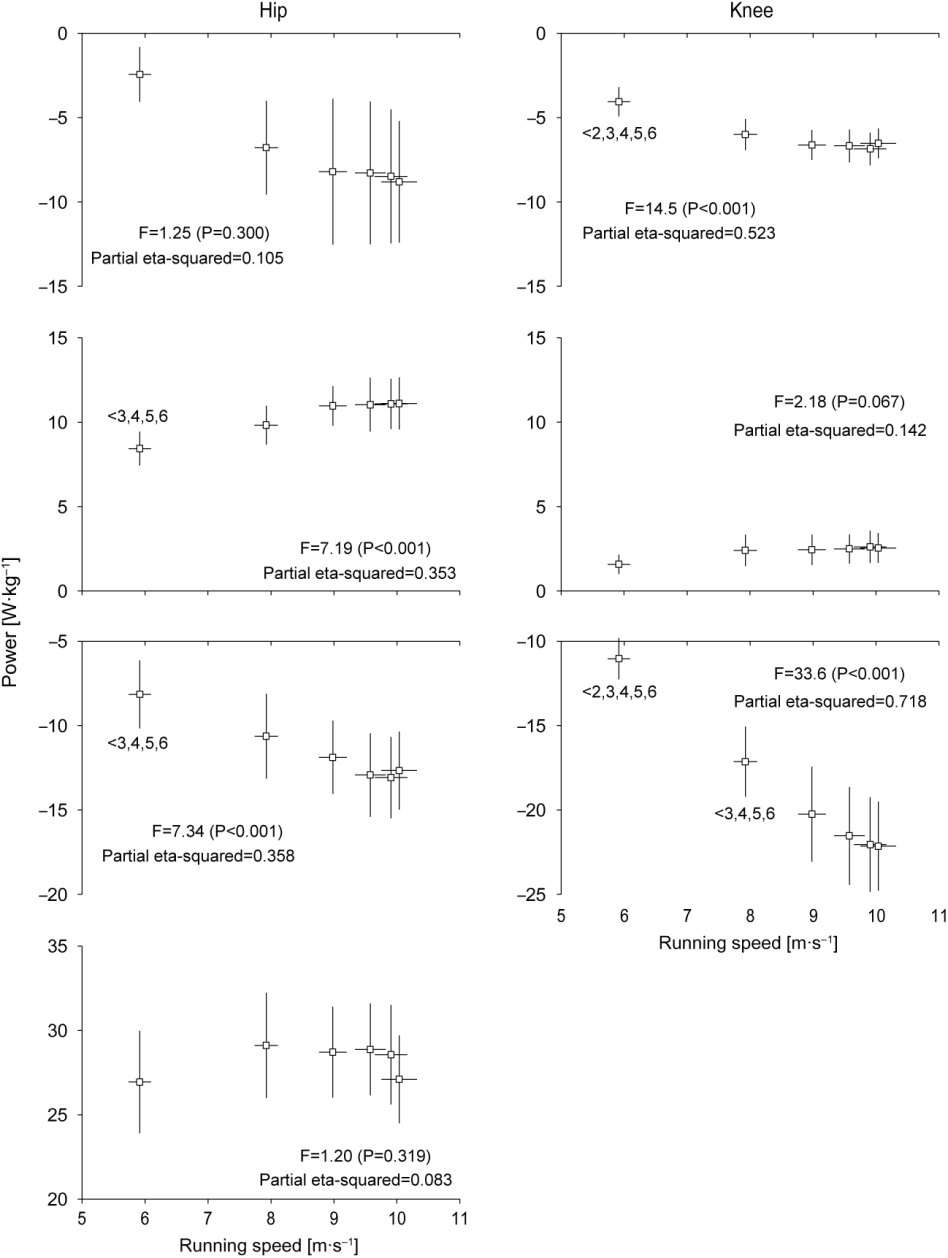
**Changes in mean power generated and absorbed by the hip and knee joints during the respective sub-phases of the entire swing phase of accelerated sprinting.** The left and right panels show 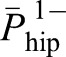 and 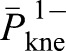, 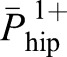 and 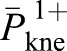, and 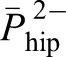 and 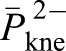, in top to the second lowest rows, respectively. The bottom left panel shows 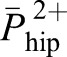. Values for all 24 swing phases were pooled into six sections (section 1 to 6) of swing phases (four swing phases per section), and these are presented as means±s.d. of 12 participants. See Materials and Methods for further detail. The inequality signs with numbers indicate the results of the Bonferroni *post hoc* test. Note the different *y*-axis scales for the panels.

Fig. 6 shows changes in mean power generated and absorbed by the hip and knee joints during the respective sub-phases of the entire swing phase in acceleration phase of sprinting. Although there was no statistically significant change in the magnitude, the first negative hip mean power (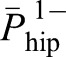) during the swing phase was initially −2.4±1.6 W kg^−1^, increased to section 3, plateaued to section 4, and increased again during the acceleration phase (−8.8±3.6 W kg^−1^ at section 6; 3.7-fold increase in total). The first positive hip mean power (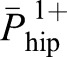) during the swing phase was initially 8.4±1.0 W kg^−1^, increased by 32% by section 3, and plateaued thereafter (11.1±1.5 W kg^−1^ at section 6). The second negative hip mean power (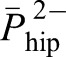) during the swing phase was initially −8.2±2.0 W kg^−1^, increased by 55% by section 5 (−12.7±2.3 W kg^−1^), and decreased subsequently. While no significant change was found, the second positive hip mean power (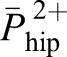) during the swing phase was initially 26.9±3.0 W kg^−1^, increased by 8% by section 2 (29.1±3.1 W kg^−1^), and decreased slightly by section 5 and greatly afterwards (27.1±2.6 W kg^−1^ at section 6). The profiles of the changes in knee joint mean power for the respective swing sub-phases were similar to those for knee joint work. The initial swing phase values were −4.1±0.9 W kg^−1^ for the first knee negative mean power (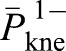), 1.6±0.6 W kg^−1^ for the first positive knee mean power (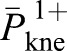), and −11.0±1.2 W kg^−1^ for the second negative knee mean power (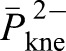). The maximal values were −6.9±1.0 W kg^−1^ at section 5 for 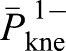, 2.6±1.0 W kg^−1^ at section 5 for 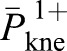 and −22.2±2.6 W kg^−1^ at section 6 for 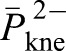.

### Association of increases in running speed with changes in joint work and power during sub-phases of the swing phase in accelerated sprinting

Table 1 shows selected joint work and mean power variables that have an influence on the increment of running speed, in each section using stepwise multiple-regression analyses. Among the joint work variables, stepwise multiple-regression analyses selected the following influences on the increment of speed during accelerated sprinting (noting that a positive joint work variable with a negative *β* value or a negative joint work variable with a positive *β* value indicates greater magnitude of positive or negative increase in variable having a negative influence on an increment of running speed): 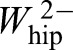 (*β*=0.848), 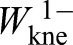 (*β*=0.819), 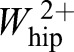 (*β*=0.674) and 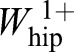 (*β*=0.596) in section 1 (adjusted *R^2^*=0.370, *P*<0.001); 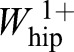 (*β*=0.641) and 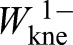 (*β*=−0.353) in section 2 (adjusted *R^2^*=0.543, *P*<0.001); 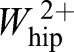 (*β*=−0.664) and 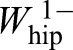 (*β*=0.503) in section 3 (adjusted *R^2^*=0.257, *P*<0.001); 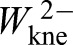 (*β*=−0.774), 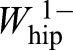 (*β*=−0.557), 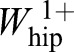 (*β*=−0.444), 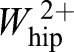 (*β*=0.437), 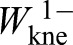 (*β*=0.326) and 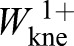 (*β*=−0.291) in section 4 (adjusted *R^2^*=0.730, *P*<0.001); 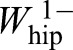 (*β*=−1.099), 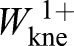 (*β*=−0.581) and 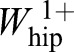 (*β*=−0.368) in section 5 (adjusted *R^2^*=0.754, *P*<0.001); and 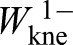 (*β*=0.885), 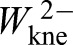 (*β*=−0.881), 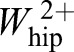 (*β*=0.555) and 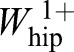 (*β*=−0.279) in section 6 (adjusted *R^2^*=0.768, *P*<0.001).

**Table 1.**
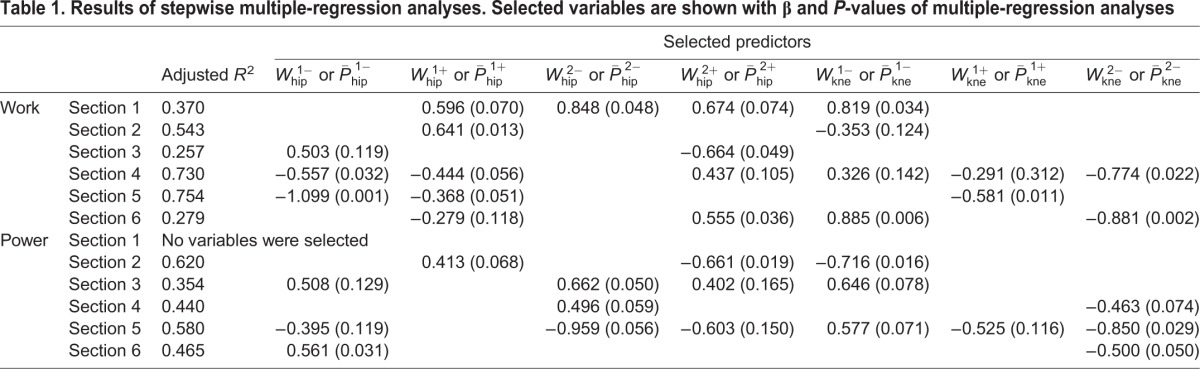
**Results of stepwise multiple-regression analyses. Selected variables are shown with β and *P*-values of multiple-regression analyses**

Among the mean power variables, stepwise multiple-regression analyses selected the following influence on the increment of speed during accelerated sprinting (noting that a positive mean power variable with a negative *β* value or a negative mean power variable with a positive *β* value indicates greater magnitude of positive or negative increase in variable having a negative influence on an increment of running speed): no variables in section 1; 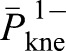 (*β*=−0.716), 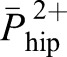 (*β*=−0.661) and 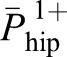 (*β*=0.413) in section 2 (adjusted *R^2^*=0.620, *P*<0.001); 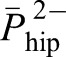 (*β*=0.662), 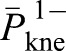 (*β*=0.646), 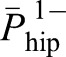 (*β*=0.508) and 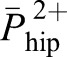 (*β*=0.402) in section 3 (adjusted *R^2^*=0.354, *P*<0.001); 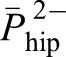 (*β*=0.496) and 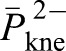 (*β*=−0.463) in section 4 (adjusted *R^2^*=0.440, *P*<0.001); 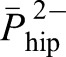 (*β*=−0.959), 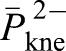 (*β*=−0.850), 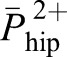 (*β*=0.603), 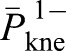 (*β*=0.577), 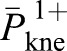 (*β*=−0.525) and 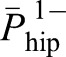 (*β*=−0.395) in section 5 (adjusted *R^2^*=0.580, *P*<0.001); and 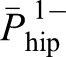 (*β*=0.561) and 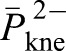 (*β*=−0.500) in section 6 (adjusted *R^2^*=0.465, *P*<0.001).

## DISCUSSION

This study demonstrated the changes in lower-extremity joint work and power during the swing phase in maximal accelerated sprinting (until 10.03±0.28 m s^−1^ at 42.7±1.9 m) and investigated whether the magnitudes of joint work and power decrease when approaching maximal speed. To the best of our knowledge, this study is the first to show that lower-extremity joint work and mean power change as running speed increases during maximal accelerated sprinting, up until maximal speed is reached. Our results provide several interesting findings: (1) 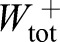 during the swing phase was initially ≈1.5-fold greater than 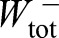, while the proportion of increment of 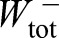 was substantially larger than that of 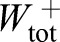 (≈2- vs 1.34-fold) with acceleration during sprinting. (2) Over half the work and power variables during the entire and respective sub-phases of the swing phase (particularly 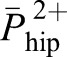) did not increase until maximal speed was reached, and most were maintained or decreased after section 4 (9.69±0.25 m s^−1^ at 26.2±1.1 m mark) or 5 (9.97±0.26 m s^−1^ at 34.3±1.5 m mark) of the acceleration phase. (3) Only the 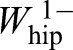, 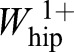, 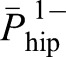 and 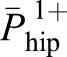, as well as 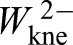 and 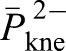, increased until maximal speed was reached. (4) In general, magnitudes of increases in positive work and mean power at hip joint during the swing phase (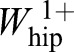, 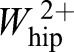, 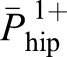 and 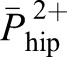) were positively associated with the increment of running speed until section 3 (18.3±0.8 m mark), while magnitudes of increases in negative work and mean power at hip and knee joints during the swing phase (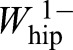, 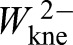, 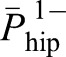 and 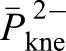) were positively associated with increased running speed subsequently. These findings indicate that the roles of energy generation and absorption at the hip and knee joints likely shift around the middle of the acceleration phase as energy generation and absorption at the hip during the late swing phase and at the knee during the early swing phase are generally maintained or decreased. Thus, the hypothesis was generally supported by the current results. Moreover, the negative hip work and mean power during the early swing phase, as well as negative knee work and mean power during the terminal swing phase, may be responsible for the final increase in running speed to maximal.

The results of this study show, using an observational approach, that the profiles of hip and knee joint moments, angular velocities and powers during each swing phase generally do not change throughout the entire acceleration phase, except for the hip flexion moment in the second swing phase, although the magnitudes change considerably (Fig. 2). The consistency of the profiles of the swing phase joint moment and power during the entire acceleration phase suggests that insofar as the swing phase, the pattern of biomechanical loads applied to the joints in one stride cycle do not change during the entire acceleration phase of maximal sprinting, except for the initial acceleration phase.

The amount of total energy absorption was small in the initial section (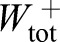 being ≈1.5-fold greater than 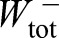), but increased with larger proportion (≈2-fold) than the amount of total energy generation (≈1.34-fold) during accelerated sprinting. The characteristics of the changes in 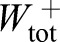 and 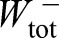 revealed in this study, that is, an increment with decrease in magnitude, are inconsistent with those in Schache et al. (2015), which investigated running at different steady speeds and found increments with a slight increase in magnitude. These differences in profiles of changes in values show the specificity of the mechanism (in terms of mechanical loads at lower-extremity joints) for increasing running speed during acceleration of sprinting. The discrepancy is presumably due to the difference in testing protocols (comparing running during acceleration versus various steady speeds). Moreover, some aspects of the differences between accelerated running and steady speed running help explain the disparity: while step frequency increases rapidly for the initial 10 m and plateaus thereafter during accelerated sprinting (Nagahara et al., 2014a,b), step frequency increases gradually then rapidly as steady running speed increases (Dorn et al., 2012; Weyand et al., 2000). If the running speed is comparable, higher step frequency may impose greater energy generation and absorption at joints during the swing phase, and this probably leads to a rapid increase in the total work done during the first half of the acceleration phase of maximal sprinting.

The magnitudes of work and power variables at hip and knee joints during the entire and sub-phases of the swing phase in section 6 of the current study were generally equivalent to those in a previous study of the maximal speed phase (Schache et al., 2011, 2015), although the values in sub-phases were overall relatively small in this study. Until running speed increased to section 4 (9.69±0.25 m s^−1^ at 26.2±1.1 m), 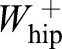 and 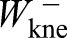 (increased ≈1.0 and ≈1.2 J kg^−1^), as well as 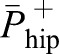 and 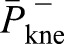 (increased ≈2.0 and ≈3.2 W kg^−1^), contributed to a similar extent to running at higher speeds in accelerated sprinting. However, from section 4 to 5 (9.97±0.26 m s^−1^ at 34.3±1.5 m), interestingly, 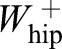 and 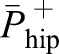 plateaued at ≈4.3 J kg^−1^ and ≈13.3 W kg^−1^, and then decreased from section 5 to 6 (10.03±0.28 m s^−1^ at 42.7±1.9 m), while 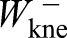 and 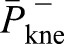 increased until section 5 (−2.88±0.22 J kg^−1^ and −5.5±0.6 W kg^−1^) and subsequently decreased. Moreover, 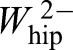, 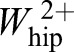 and 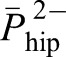 (−0.75±0.15 J kg^−1^, 2.78±0.28 J kg^−1^ and −12.93±2.47 W kg^−1^ at section 4), as well as 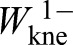 and 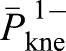 (−0.93±0.11 J kg^−1^ and −6.68±0.97 W kg^−1^ at section 4), plateaued or decreased after section 4. Furthermore, stepwise multiple-regression analyses revealed that the important role of the swing leg joints for increasing running speed during accelerated sprinting generally shifted from energy generations during the first half (until section 3) to energy absorptions during the second half, although there were some exceptions. These findings demonstrate that the role of the respective joints in increasing running speed during acceleration shifts at section 4, and explain the background of previous findings as the magnitudes of mean angular velocity during the support phase and range of flexion/extension of hip joint gradually and slightly decrease from the 14th step (Nagahara et al., 2014a).

Although it is difficult to conclude the reason for the absence of the increase in joint work and power variables during later acceleration phase especially in positive values, some backgrounds can be explained in reference to previous studies. There is a concept of the force-velocity relationship of muscles, i.e. the muscle can produce greater contraction force at slower contraction speed and the magnitude of the force production reaches maximum under eccentric force production condition (Lieber, 1992). While the step duration (inverse of step frequency) is fairly constant throughout the acceleration phase of sprinting except for initial four or five steps (Nagahara et al., 2014a), the swing velocities of entire lower-extremity in forward and backwards direction increase with increase in running speed, because the distal end point of that have to exceed the running speed for acceleration. Moreover, hip and knee are responsible for decelerating lower-extremity before the ends of both forward and backwards swing during sprinting, suggesting that those joints need to eccentrically produce the power especially at higher speeds. Therefore, it is reasonable that the increment of energy generation with concentric force production become difficult at higher running speeds during accelerated sprinting and the increase in energy absorption become dominant for the increment of total energy. Around the 14th step (section 4 in this study), changes in trunk posture terminate and the magnitude of hip flexion/extension range start to decrease slightly (Nagahara et al., 2014a). The energy would be generated to raise the trunk from forward hanging to upright posture. Thus, the end of raising the trunk and the decrement of the demand to swing the legs widely forward and backwards would suppress the increase in joint work and mean power during the swing phase from section 4 of the acceleration phase.


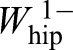
, 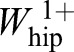 and 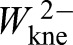 (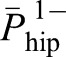 and 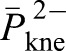) only increased when running speed increased between sections 4 and 6 [29.6%, 2.2% and 4.3% (6.3% and 2.9%)]. Moreover, stepwise multiple-regression analyses verified that generally the energy absorption at the hip (during the early swing phase) and knee joints (during the terminal swing phase) of the swing leg became important for effective acceleration from section 4 (9.69±0.25 m s^−1^ at 26.2±1.1 m). Thus, energy absorption and generation at the hip during the early swing phase and energy absorption at the knee during the terminal swing phase may play important roles in increasing running speed from nearly maximum to maximum during accelerated sprinting. Nagahara et al. (2014a) speculated that the stable upright trunk posture from the 14th step during accelerated sprinting leads to increased muscle tension in the front of the body, particularly the iliopsoas muscle, and sprinters can then swing the leg forward more rapidly (Dorn et al., 2012) with a small range of motion, possibly along with reduced hip extension velocity before the toe-off. The increment of 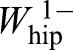 and 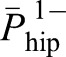 after section 4 of accelerated sprinting would partially support this speculation. Moreover, interestingly, the 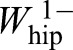 and 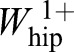 of one leg during the early swing phase occurred simultaneously with the 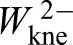 of the other leg during the late swing phase, that is, power production and absorption occur at approximately the same time (Fig. 1). Faster backwards or forward swing of the leg cannot be accomplished by a single leg during the flight phase, and the counteracting swing movement of the other leg is requisite. Moreover, it seems that the increase in iliopsoas muscle tension of one leg during the early swing phase, tilting the pelvis forward, induces substantial hamstring stretch in the opposite limb (Chumanov et al., 2007). Consequently, simultaneous energy absorption and generation by the hip and absorption by the contralateral knee may develop synergistically after section 4 (26.2±1.1 m), and this interaction would be partially responsible for the increase in running speed until the maximal speed is reached.

### Limitations

The major limitation of the present study is that the variables were only investigated during the swing phase of sprinting, because there were no force platforms for collecting ground reaction forces during the entire acceleration phase of sprinting. Although the information obtained during the swing phase helps the understanding of maximal accelerated locomotion of humans, investigation of lower-extremity work and power during the support phase provides a deeper understanding as the body is horizontally propelled or braked only during the support phase (ignoring air resistance). Therefore, this is an area for future investigation. Second, the data obtained in this study were from relatively homogeneous participants only including male sprinters. Sprinter's acceleration shows characteristic features, e.g. deep forward inclination of a trunk and the entire body during the initial steps in contrast to other sprinting athletes such as soccer players. Thus, when an investigation of joint kinetics with other cohorts is performed, different profiles of changes in work and power during accelerated sprinting are likely to be found. That said, sprinters have developed their capability to maximize acceleration performance, and thus the findings from them could be normative.

### Conclusions

The results of the current study indicate that the roles of the hip and knee joints during the swing phase shift around the middle of the acceleration phase as energy generation and absorption at the hip during the late swing phase and at the knee during early swing phase are maintained or decreased (especially, the second power generation by the hip during the swing phase). Energy absorption at the hip during the early swing phase and at the knee during the terminal swing phase are probably responsible for increasing running speed when approaching maximal speed (after reaching 9.19±0.23 m s^−1^ at 18.3±0.8 m). These findings would allow us to understand the function of hip and knee joints during swing phase and its change as a part of a locomotor system in sprinting under accelerated condition.

## MATERIALS AND METHODS

### Participants

Twelve male sprinters participated in this study (mean±s.d.: age, 21.6±2.6 years; height, 1.74±0.04 m; body mass, 68.1±4.2 kg; personal best 100 m race time, 10.71±0.33 s), having provided written informed consent. This study was approved by the ethics committee of the University of Tsukuba.

### Procedure

After their regular warm-up, each participant sprinted for 60 m, wearing spiked shoes, twice with maximal effort from their crouched starting position. Three-dimensional coordinate data from 47 retro-reflective markers affixed to the participant's body were collected with 60 infrared cameras (Vicon Motion Systems, Oxford, UK; 250 Hz) as described in previous studies (Nagahara et al., 2014a; Suzuki et al., 2014). The captured volume was approximately 50 m×1.5 m×2 m (length×width×height). The 60 m sprint time was recorded using a photocell system (HL2-35, Tag Heuer, La Chaux-de-Fonds, Switzerland).

### Data processing and analysis

The three-dimensional marker coordinates from the fastest 60 m sprint trial (determined by the 60 m sprint time) for each participant were analyzed. Endpoints of 15 segments of the whole body, consisting of head, upper trunk, lower trunk, hands, forearms, upper arms, feet, shanks, and thighs, were determined using the marker coordinates in accordance with previous studies (Nagahara et al., 2014a; Suzuki et al., 2014). The endpoint coordinates were smoothed with a fourth-order Butterworth low-pass digital filter. The cut-off frequency was 12 Hz (Chumanov et al., 2011; Debaere et al., 2013). Foot strike and toe-off for all steps were determined by vertical acceleration and the positions of markers on the toes using previously proposed kinematic data based methods (Hreljac and Marshall, 2000; Nagahara and Zushi, 2013).

**Fig. 7.**
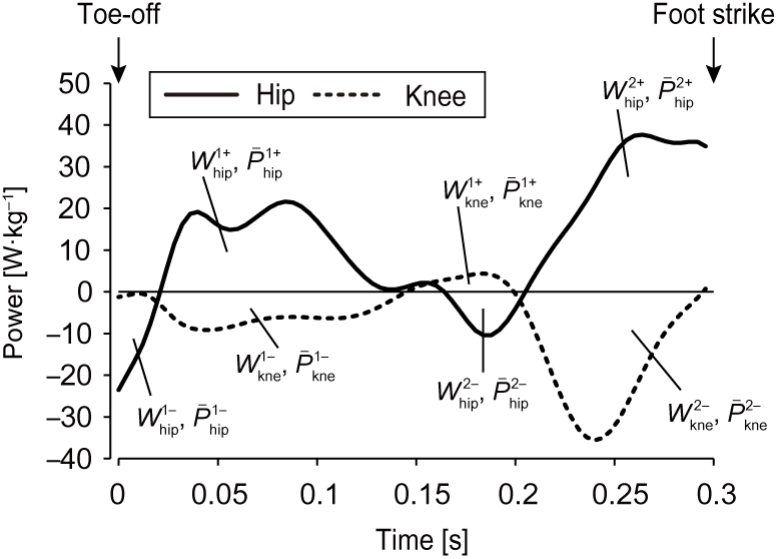
**Example of the sub-phases for hip and knee work and mean power during one swing phase.**

Joint moments at the hip, knee and ankle during the swing phase were calculated using a standard inverse-dynamics analysis for both legs (Winter, 2009). The moments applied around segmental centers of mass were initially calculated by differentiating each segment's angular momentum in the global reference frame. Subsequently, in accordance with Robertson et al. (2004), joint moments during the swing phase were computed from the lower-extremity kinematics and body segment inertia properties based on analysis of free-body-diagrams for each segment. The location of the center of mass and the inertia parameters of the respective segments were estimated from the body segment parameters of Japanese athletes (Ae, 1996). Joint power during the swing phase was calculated by the dot product of joint moment and angular velocity at each joint (Rubenson et al., 2011; Schache et al., 2011, 2015). The positive and negative works at joints were computed by integrating the joint powers over the duration of the swing phase. The 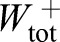 or 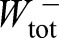 was calculated as the sum of positive or negative work done at the hip (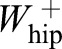, 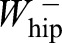), knee (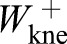, 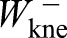) and ankle joints. The positive and negative mean power at joints during the swing phase were determined by dividing the positive and negative work at each joint by the corresponding swing time (Farris and Sawicki, 2012; Schache et al., 2015). As shown in Fig. 7, the swing phase was divided into four and three sub-phases for hip and knee, respectively, in accordance with the phase profiles of hip and knee joint power described in a previous study (Schache et al., 2011). The joint work and mean power during the respective sub-phases were calculated. All variables for each participant were normalized to body mass. To illustrate typical alterations, joint moments, angular velocities and powers for all participants were time normalized as a percentage of the respective swing phases. As coordinate data were only obtained until the 25th step, variables from the first swing phase (from the toe-off of the 1st step, not the swing phase just after block clearance) to the swing phase after the 24th step (around the maximal speed) were able to be analyzed.

### Statistics

Means±s.d. of the time-normalized joint moments, angular velocities and powers during the swing phase, and of the works and mean powers during swing phase, were calculated. The Shapiro–Wilk test was used to test normality of the data. Changes in variables during accelerated sprinting were evaluated by one-way analysis of variance (ANOVA) with repeated measures. To investigate the overall changes in values during the acceleration phase, values for all 24 swing phases were pooled into six sections (section 1 to 6) of swing phases (four swing phases per section) for analysis by ANOVA. For each ANOVA, partial eta-squared was calculated as a measure of effect size. When a significant difference was detected, data were analyzed using the Bonferroni post-hoc test. In addition, associations of an increase in running speed (dependent variables) with changes in joint work and power during respective sub-phases (seven independent variables) in each section were tested with stepwise-multiple-regression analysis using the step-up procedure and Bayesian information criterion. Effects of changes in joint works or powers on increase in running speed were examined separately in order to avoid an influence of the multicollinearity of variables. Increased or decreased amounts of variables in each section were obtained as differences in values between two consecutive sections (e.g. subtracting the values in section 1 from the corresponding values in section 2 as the increased amounts of values in section 2) except for section 1. Values in section 1 (not deltas) were used as the increased amounts of variables for the analysis of section 1. JMP 12 statistical software (SAS Institute Japan Ltd, Tokyo, Japan) was used to calculate all statistical values except the partial eta-squared, which was calculated by dividing the specific sum of squares by the total sum of squares. Statistical significance was set at *P*<0.05.

## References

[BIO024281C1] Ae M. (1996). Body segment inertia parameters for Japanese children and athletes. *Jpn. J. Sports Sci.* 15, 155-162.

[BIO024281C2] Ae M., Miyashita K., Yokoi T. and Hashihara Y. (1987). Mechanical power and work done by the muscles of the lower limb during running at different speeds. In *Biomechanics X-B*, Vol. 6B (ed. Jonsson B.), pp. 895-899. Champaign, IL: Human Kinetics Publishers, Inc.

[BIO024281C3] Belli A., Kyröläinen H. and Komi P. V. (2002). Moment and power of lower limb joints in running. *Int. J. Sports Med.* 23, 136-141. 10.1055/s-2002-20136 11842362

[BIO024281C4] Biewener A. A. and Daley M. A. (2007). Unsteady locomotion: integrating muscle function with whole body dynamics and neuromuscular control. *J. Exp. Biol.* 210, 2949-2960. 10.1242/jeb.005801 17704070 PMC2651961

[BIO024281C5] Chapman A. E. and Caldwell G. E. (1983). Kinetic limitations of maximal sprinting speed. *J. Biomech.* 16, 79-83. 10.1016/0021-9290(83)90048-9 6833312

[BIO024281C6] Chumanov E. S., Heiderscheit B. C. and Thelen D. G. (2007). The effect of speed and influence of individual muscles on hamstring mechanics during the swing phase of sprinting. *J. Biomech.* 40, 3555-3562. 10.1016/j.jbiomech.2007.05.026 17659291

[BIO024281C7] Chumanov E. S., Heiderscheit B. C. and Thelen D. G. (2011). Hamstring musculotendon dynamics during stance and swing phases of high-speed running. *Med. Sci. Sports Exerc.* 43, 525-532. 10.1249/MSS.0b013e3181f23fe8 20689454 PMC3057086

[BIO024281C8] Debaere S., Delecluse C., Aerenhouts D., Hagman F. and Jonkers I. (2013). From block clearance to sprint running: characteristics underlying an effective transition. *J. Sports Sci.* 31, 137-149. 10.1080/02640414.2012.722225 22974278

[BIO024281C9] Dorn T. W., Schache A. G. and Pandy M. G. (2012). Muscular strategy shift in human running: dependence of running speed on hip and ankle muscle performance. *J. Exp. Biol.* 215, 1944-1956. 10.1242/jeb.064527 22573774

[BIO024281C10] Farris D. J. and Sawicki G. S. (2012). The mechanics and energetics of human walking and running: a joint level perspective. *J. R. Soc. Interface* 9, 110-118. 10.1098/rsif.2011.0182 21613286 PMC3223624

[BIO024281C11] Hreljac A. and Marshall R. N. (2000). Algorithms to determine event timing during normal walking using kinematic data. *J. Biomech.* 33, 783-786. 10.1016/S0021-9290(00)00014-2 10808002

[BIO024281C12] Hunter J. P., Marshall R. N. and McNair P. J. (2004). Interaction of step length and step rate during sprint running. *Med. Sci. Sports Exerc.* 36, 261-271. 10.1249/01.MSS.0000113664.15777.53 14767249

[BIO024281C13] Johnson M. D. and Buckley J. G. (2001). Muscle power patterns in the mid-acceleration phase of sprinting. *J. Sports Sci.* 19, 263-272. 10.1080/026404101750158330 11311024

[BIO024281C14] Knuesel H., Geyer H. and Seyfarth A. (2005). Influence of swing leg movement on running stability. *Hum. Mov. Sci.* 24, 532-543. 10.1016/j.humov.2005.08.002 16213046

[BIO024281C15] Lieber R. (1992). *Skeletal Muscle Structure and Function: Implications for Rehabilitation and Sports Medicine*. Baltimore, MD: Williams & Wilkins.

[BIO024281C16] McGowan C. P., Baudinette R. V. and Biewener A. A. (2005). Joint work and power associated with acceleration and deceleration in tammar wallabies (Macropus eugenii). *J. Exp. Biol.* 208, 41-53. 10.1242/jeb.01305 15601876

[BIO024281C17] Morin J.-B., Slawinski J., Dorel S., Saez-de-Villarreal E., Couturier A., Samozino P., Brughelli M. and Rabita G. (2015). Acceleration capability in elite sprinters and ground impulse: Push more, brake less? *J. Biomech.* 48, 3149-3154. 10.1016/j.jbiomech.2015.07.009 26209876

[BIO024281C18] Nagahara R. and Zushi K. (2013). Determination of foot strike and toe-off event timing during maximal sprint using kinematic data. *Int. J. Sport. Health Sci.* 11, 96-100. 10.5432/ijshs.201318

[BIO024281C19] Nagahara R., Matsubayashi T., Matsuo A. and Zushi K. (2014a). Kinematics of transition during human accelerated sprinting. *Biol. Open* 3, 689-699. 10.1242/bio.20148284 24996923 PMC4133722

[BIO024281C20] Nagahara R., Naito H., Morin J.-B. and Zushi K. (2014b). Association of acceleration with spatiotemporal variables in maximal sprinting. *Int. J. Sports Med.* 35, 755-761. 10.1055/s-0033-1363252 24577864

[BIO024281C21] Novacheck T. F. (1998). The biomechanics of running. *Gait Posture* 7, 77-95. 10.1016/S0966-6362(97)00038-6 10200378

[BIO024281C22] Rabita G., Dorel S., Slawinski J., Sàez-de-Villarreal E., Couturier A., Samozino P. and Morin J.-B. (2015). Sprint mechanics in world-class athletes: a new insight into the limits of human locomotion. *Scand. J. Med. Sci. Sports* 25, 583-594. 10.1111/sms.12389 25640466

[BIO024281C23] Robertson D. G. E., Caldwell G. E., Hamill J., Kamen G. and Whittlesey S. N. (2004). *Research Methods in Biomechanics*. Champaign, IL: Human Kinetics Publishers, Inc.

[BIO024281C24] Rubenson J. and Marsh R. L. (2009). Mechanical efficiency of limb swing during walking and running in guinea fowl (Numida meleagris). *J. Appl. Physiol.* 106, 1618-1630. 10.1152/japplphysiol.91115.2008 19228989 PMC2681329

[BIO024281C25] Rubenson J., Lloyd D. G., Heliams D. B., Besier T. F. and Fournier P. A. (2011). Adaptations for economical bipedal running: the effect of limb structure on three-dimensional joint mechanics. *J. R. Soc. Interface* 8, 740-755. 10.1098/rsif.2010.0466 21030429 PMC3061092

[BIO024281C26] Schache A. G., Blanch P. D., Dorn T. W., Brown N. A., Rosemond D. and Pandy M. G. (2011). Effect of running speed on lower limb joint kinetics. *Med. Sci. Sports Exerc.* 43, 1260-1271. 10.1249/MSS.0b013e3182084929 21131859

[BIO024281C27] Schache A. G., Brown N. A. T. and Pandy M. G. (2015). Modulation of work and power by the human lower-limb joints with increasing steady-state locomotion speed. *J. Exp. Biol.* 218, 2472-2481. 10.1242/jeb.119156 26056240

[BIO024281C28] Segers V., Aerts P., Lenoir M. and De Clerq D. (2007). Dynamics of the body centre of mass during actual acceleration across transition speed. *J. Exp. Biol.* 210, 578-585. 10.1242/jeb.02693 17267643

[BIO024281C29] Segers V., Van Caekenberghe I., De Clercq D. and Aerts P. (2014). Kinematics and dynamics of burst transitions. *J. Mot. Behav.* 46, 267-276. 10.1080/00222895.2014.896780 24773232

[BIO024281C30] Suzuki Y., Ae M., Takenaka S. and Fujii N. (2014). Comparison of support leg kinetics between side-step and cross-step cutting techniques. *Sports Biomech.*. 13, 144-153. 10.1080/14763141.2014.910264 25122999

[BIO024281C31] Van Caekenberghe I., Segers V., Willems P., Gosseye T., Aerts P. and De Clercq D. (2013). Mechanics of overground accelerated running vs. running on an accelerated treadmill. *Gait Posture* 38, 125-131. 10.1016/j.gaitpost.2012.10.022 23228623

[BIO024281C32] Vardaxis V. and Hoshizaki T. B. (1989). Power patterns of the leg during the recovery phase of the sprinting stride for advanced and intermediate sprinters. *Int. J. Sport Biomech.* 5, 332-349. 10.1123/ijsb.5.3.332

[BIO024281C33] Weyand P. G., Sternlight D. B., Bellizzi M. J. and Wright S. (2000). Faster top running speeds are achieved with greater ground forces not more rapid leg movements. *J. Appl. Physiol.* 89, 1991-1999. 11053354 10.1152/jappl.2000.89.5.1991

[BIO024281C34] Winter D. A. (2009). *Biomechanics and Motor Control of Human Movement*. New York, NY: John Wiley & Sons, Inc 10.1002/9780470549148

